# Applying a new theory to smoking cessation: case of multi-theory model (MTM) for health behavior change

**DOI:** 10.15171/hpp.2017.18

**Published:** 2017-03-05

**Authors:** Manoj Sharma, Jagdish Khubchandani, Vinayak K. Nahar

**Affiliations:** ^1^Behavioral & Environmental Health, School of Public Health, Jackson State University, Jackson, MS, USA; ^2^College of Health Sciences, Walden University, Minneapolis, MN, USA; ^3^Department of Physiology and Health Science, Ball State University, Muncie, IN, USA; ^4^Department of Health, Physical Education, and Exercise Science, Lincoln Memorial University, Harrogate, TN, USA

**Keywords:** Smoking, Tobacco use, Multi-theory model, Health behavior change, Intervention, Questionnaire

## Abstract

**Background:** Smoking continues to be a public health problem worldwide. Smoking and tobacco use are associated with cardiovascular diseases that include coronary heart disease, atherosclerosis, cerebrovascular disease, and abdominal aortic aneurysm. Programs for quitting smoking have played a significant role in reduction of smoking in the United States. The smoking cessation interventions include counseling, nicotine replacement therapy, buproprion therapy, and varenicline therapy. The success rates with each of these approaches vary with clear need for improvement. Moreover, there is a need for a robust theory that can guide smoking cessation counseling interventions and increase the success rates. A fourth generation approach using multi-theory model (MTM) of health behavior change is introduced in this article for smoking cessation. An approach for developing and evaluating an intervention for smoking cessation is presented along with a measurement tool.

**Methods:** A literature review reifying the MTM of health behavior change for smoking cessation has been presented. An instrument designed to measure constructs of MTM and associated smoking cessation behavior has been developed.

**Results: ** The instrument developed is available for validation, reliability and prediction study pertaining to smoking cessation. The intervention is available for testing in a randomized control trial involving smokers.

**Conclusion:** MTM is a robust theory that holds promise for testing and application to smoking cessation.

## Introduction

Tobacco use and cigarette smoking are a major preventable public health problem globally. According to the World Health Organization (WHO) in 2012, 21% of the total world population above 15 years smoked tobacco.^1^ In the United States from 1965 to 2010, the prevalence of cigarette smoking among adults has decreased from 42.4% to 17.8% but is still a major public health issue.^2,3^ In the United States, data from the 2014 National Youth Tobacco Survey (NYTS) estimated that 4.6 million middle and high school students were current users of tobacco and out of these an estimated 2.2 million were current users of two or more types of tobacco products.^4^

Smoking and tobacco use are associated with a number of negative effects. They are associated with cardiovascular diseases that include coronary heart disease, atherosclerosis, cerebrovascular disease and abdominal aortic aneurysm.^5^ Tobacco is also a causative agent for several cancers including those of lung, lip, mouth, pharynx, esophagus, stomach, pancreas, larynx, trachea, cervix, kidney, bladder and acute myeloid leukemia.^6^ Tobacco is also associated with respiratory diseases that include chronic obstructive pulmonary disease (COPD), pneumonia, decreased lung function, and asthma related symptoms; reproductive problems such as reduced fertility, fetal death, still birth, low birth weight, and pregnancy complications; and other diseases such as sudden infant death syndrome (SIDS), cataracts, adverse surgical outcomes, low bone density, and hip fractures.^5^

Quitting smoking is advantageous for health especially for smokers who quit before the age of 35 years as their mortality rates are similar to those who have never smoked.^3^ Programs for quitting smoking have played a significant role in reduction of smoking in the United States. In 2010, the Centers for Disease Control and Prevention (CDC) reported that 68.8% of adult smokers wanted to stop smoking and 52.4% had made a quit attempt in the past year but only 6.2% had been successful in quitting.^3^ The smoking cessation interventions include counseling, nicotine replacement therapy, buproprion therapy, and varenicline therapy.^7^ The success rates with each of these approaches vary with clear need for improvement. In counseling various methods are popular that include telephone counseling, interactive computer programs, training of health care providers such as physicians or pharmacists and counseling by lay health volunteers. One of the theories that has guided such counseling efforts is the transtheoretical model.^8,9^ The transtheoretical model or, sometimes also called as, the stages of change model proposes that smokers move through a series of discrete stages before they quit successfully namely precontemplation (no plans of quitting), contemplation (planning to quit), preparation (planning to quit within the next 30 days), action (successful quitting for up to six months), and maintenance (abstinence for more than six months). In 2010, a meta-analysis was done that compared stage-based interventions with non-stage-based controls and it concluded that stage-based counseling programs were neither more nor less effective than their non-stage-based equivalents.^10^ So this emphasizes the need for a more robust theory that can guide smoking cessation counseling interventions and increase the success rates. Using the same approach that Prochaska used for developing his theory in 1979,^8^ after reviewing existing theories, Sharma has developed the first theory that is by a health education specialist and is exclusive to health behavior change.^11^ It is believed that this theory if implemented can improve the success rate of smoking cessation programs. The purpose of this article to present a tool that can be used to measure the changes in the constructs of this theory and also present a framework for an intervention.

## Materials and Methods

### Multi-theory model of health behavior change

This theory uses successful and empirically tested constructs from existing theories, attempts to be parsimonious, attempts to address health behavior change as opposed to a mere acquisition of a behavior, avoids overlap among constructs so that there is no shared variance, addresses both immediate and long term change, attempts to be culturally viable and also appropriate for resource scarce settings.^9,11^ Previous studies have demonstrated empirical evidence supporting predictive ability of MTM with several health behaviors such as physical activity, small portion size consumption, and adequate sleep.^12-14^ This theory dissects health behavior *change* into two components: initiation of the behavior change and sustenance or continuation of the health behavior change. In the context of smoking cessation initiation would entail starting with the decision to quit smoking and sustenance would entail attaining abstinence. Why this differentiation is needed is because according to this theory the constructs that influence initiation of change are different than the constructs that sustain the behavior change.

According to this theory the first construct for initiation of smoking cessation is participatory dialogue. This has been adopted from the derived from the Freire’s model of adult education.^15^ It entails having a two-way communication between the person facilitating smoking cessation (counselor, health educator, certified health education specialist, lay health volunteer, physician, pharmacist and so on) and the person wanting to quit in which the facilitator tries to underscore the advantages of quitting over the disadvantages. This construct is also present in other models such as transtheoretical,^8^ health belief model and so on but what is different here is the two-way communication which is unique in the Freirian model.^15,16^ Some of the potential advantages and disadvantages of quitting smoking can be seen in Supplementary file 1 that depicts an instrument based on this theory that can be used for predictive modeling as well as for impact evaluation of interventions in individual, group and community settings.

**Figure 1:**
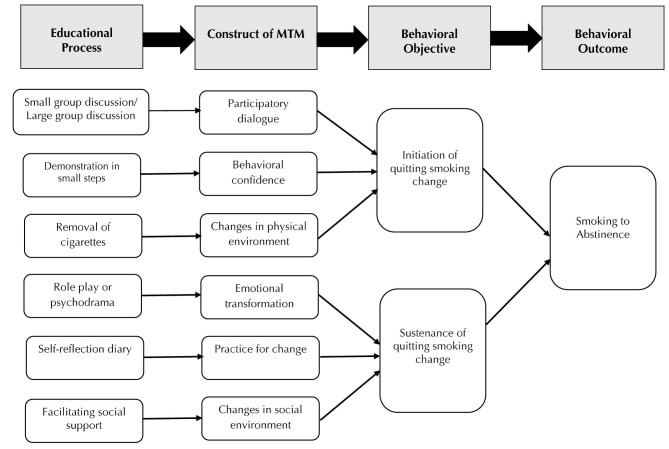
Diagrammatic depiction of how multi-theory model (MTM) for health behavior change can be used for smoking cessation in adults.

A diagrammatic depiction of this model for influencing smoking cessation in adults is shown in Figure 1. In this Figure it can be seen that participatory dialogue can be facilitated by small group discussion or large group discussion in a group or community setting. For individual settings this can be done one-on-one.

The second construct for initiation of smoking cessation according to this model is “behavioral confidence” derived from Bandura’s self-efficacy and Ajzen’s perceived behavioral control but a little different in the sense that it is futuristic as opposed to the existing conceptualization of “here and now.”^17,18^ It has been reified for smoking cessation as being able to quit this week, ability to be able to do all tasks despite quitting, ability to relax despite quitting, ability to get by without getting anxious or facing any withdrawal symptoms. In an intervention this can be reified by demonstration of quitting in small steps, attributing sources for quitting to self and influential others in life, and focusing on a quit date.

The third construct for initiation of smoking cessation is physical environment derived from Bandura’ social cognitive theory and several other theories.^18^ This has been reified as ability to get rid of all cigarettes from the environment, ability to refrain from buying cigarettes, and ability to substitute smoking time with something else. Changes in these three constructs by way of an intervention at individual, group or community level can help in initiation of quitting in smokers.

In order to sustain the behavior, change of continuing to abstain from cigarettes the first construct that is important is emotional transformation derived from the emotional intelligence theory.^9,19,20^ It has been reified for smoking cessation as the ability to direct one’s emotions/feelings to the goal of being smoke free, motivate oneself to be smoke free, and overcome self-doubt in accomplishing the goal of being smoke free. In an educational intervention it can be facilitated by conducting a role play or a psychodrama.

The second construct for sustenance of being abstinent is practice for change derived from Freire’s adult education model’s praxis which refers to active reflection and reflective action.^15^ For smoking cessation it involves keeping a self-diary to monitor one’s smoking urge, ability to be smoke free even if one encounters barriers, and ability to change one’s plans if faced with difficulties. In an educational intervention it can be developed by asking the participants to maintain a diary or a journal or in this age of technology by using apps.

The final construct for sustenance of quitting smoking is the social environment derived from construct of environment,^18^ helping relationships,^8^ social support and so on.^21^ For smoking cessation it can entail getting help from family member, friends, or health care professionals.

## Results

The first type of study that can be undertaken using the information presented in this article is to conduct validation, reliability testing and predictive study using the instrument developed in this study. For this purpose, a cross sectional study will be appropriate. The sample size for such a study using G*Power at an alpha of 0.05, power of 0.80, an estimated effect size of 0.10 (medium) and number of predictors in regression being three would come to be 114 participants.^22^ For face and content validation the development of this scale by three researchers who are the authors of this study and are adept in instrument development, one of the authors being the originator of the theory and all of them being well versed with the target population coupled with the review process entailing three blind reviewers should be sufficient. For construct validations the researchers can use either the confirmatory factor analysis process described by Sharma and Petosa or structure equation modeling.^23^ For reliability testing measuring internal consistency can be done by calculating Cronbach’s alpha.^23^ For calculating test retest reliability the scale can be administered twice to a group of 30 participants at an interval of one week and correlation coefficients calculated. For predictive modeling using the same sample of 114 participants, regression models for both initiation and sustenance can be developed.

The second type of study that can be done using the material presented in this article is an intervention study. The design for such a study can be as simple as pre-test post-test design or more sophisticated like a randomized controlled trial (RCT). For a RCT with an alpha of 0.05, power of 0.80, effect size of 0.30, two groups and three measurements the total sample size using G*Power comes to 62 or 31 in each group. The method of data analysis would be repeated measures analysis of variance (ANOVA).

## Discussion

The purpose of this article was to present a fourth generation approach using MTM of health behavior change for creating an instrument and then developing and evaluating an intervention for smoking cessation. The instrument has the potential for testing in validation studies and predictive studies using MTM as an approach. The methodology for instrument development delineated in this study has the potential to be replicated with other behaviors as well. The article also presents an approach for developing and testing a smoking cessation intervention based on MTM. If tested using a RCT it can strengthen evidence-based practice.

There are several limitations of this article. First, empirical data has not been presented in this article and is only in a protocol format. Future research would have to be undertaken to validate the tool and the intervention. Second, the tool depends on self-report as the major method for data collection. Self-reports have the potential for bias due to false reporting, dishonesty, inaccurate assessment, subjectivity etc. Researchers using this approach must also supplement measurement by more objective tools such as measurement of cotinine levels in the blood. Finally, the intervention presented in this article is only in the framework format and more details about the content, process and time allocation would need to be worked out before actual implementation of the intervention.

## Conclusion

The instrument and the framework are the starting point in any research endeavor. The tool and the framework of the intervention presented in this article would be quite beneficial to future smoking cessation researchers who can test these through their work.

## Ethical approval

Not applicable.

## Competing interests

None.

## Supplementary Materials

Online Supplementary file 1 contains an instrument for measuring change in smoking.
