# Supplementary material for: Applying a new theory to smoking cessation: case of multi-theory model (MTM) for health behavior change
Source: Health Promot Perspect. 2017 Mar 5;7(2):102–5. doi: 10.15171/hpp.2017.18 (PMC5350547; doi:10.15171/hpp.2017.18)
Supplement: Supplementary file 1 [file hpp-7-102-S001.pdf]

SUPPLEMENT 1. INSTRUMENT FOR MEASURING CHANGE IN SMOKING

Directions: This survey is voluntary, which means you may choose not to complete it or not to answer individual questions. There is no direct benefit of this survey to you. All data from this survey will be anonymous and kept secret. Your responses will help in developing effective smoking cessation programs. Please put an X mark by the response or fill the response that correctly describes your position. Thank you for your help!

1. During the past seven days, did you smoke one or more cigarettes?

- ☐ No
- ☐ Yes

If your answer is no, then you can stop taking this questionnaire. Thank you for your time

……………………………………………………………………………………………………

2. What is your gender?
- ☐ Male
- ☐ Female
- ☐ Other, ________________

……………………………………………………………………………………………………

3. How old are you today? _______ years

……………………………………………………………………………………………………

4. What is your race/ethnicity?
- ☐ White or Caucasian American
- ☐ Black or African American
- ☐ Asian American
- ☐ American Indian
- ☐ Hispanic American
- ☐ Other _________________

……………………………………………………………………………………………………

5. What is your education?
- ☐ Some schooling but not completed high school
- ☐ Completed high school or GED
- ☐ Some college
- ☐ Completed college/ Graduate degree
- ☐ Post graduate degree
- ☐ Professional degree

……………………………………………………………………………………………………

6. Do you work?
- ☐ No
- ☐ Yes, _____ hours/week (put a single number not a range)

……………………………………………………………………………………………………

7. What is your yearly household income?
- ☐ Less than $ 50,000
- ☐ $ 50,000 to $ 100,000
- ☐ $100,001 to $150,000
- ☐ $150,001 to $200,000
- ☐ More than $200,000

……………………………………………………………………………………………………

8. How long have you smoked? _____________ (in years)

……………………………………………………………………………………………………

9. How many cigarettes a day do you smoke? ________

……………………………………………………………………………………………………

	Never	Hardly Ever	Sometimes	Almost Always	Always
Participatory dialogue: Advantages					
**If you quit smoking you will…**					
10. … be healthy.	☐	☐	☐	☐	☐
11. …save money.	☐	☐	☐	☐	☐
12. … get sick less often.	☐	☐	☐	☐	☐
13. …smell better.	☐	☐	☐	☐	☐
14. … enjoy life more.	☐	☐	☐	☐	☐

	Never	Hardly Ever	Sometimes	Almost Always	Always
Participatory Dialogue: Disadvantages					
**If you quit smoking you will…**					
15. … not be able to relax as well.	☐	☐	☐	☐	☐
16. … not be able to socialize as well.	☐	☐	☐	☐	☐
17. … miss it.	☐	☐	☐	☐	☐
18. … not be able to overcome the urge.	☐	☐	☐	☐	☐
19. … lose friends.	☐	☐	☐	☐	☐

	Not At All Sure	Slightly Sure	Moderately Sure	Very Sure	Completely Sure
Behavioral confidence					
**How sure are you that you will be able to quit smoking …**					
20. … this week?	☐	☐	☐	☐	☐

	Not At All Sure	Slightly Sure	Moderately Sure	Very Sure	Completely Sure
Behavioral confidence					
**How sure are you that you will be able to quit smoking …**					
21. … this week and complete all work-related tasks?	☐	☐	☐	☐	☐
22. … this week and relax?	☐	☐	☐	☐	☐
23. … this week without getting anxious?	☐	☐	☐	☐	☐
24. … this week without getting withdrawal symptoms?	☐	☐	☐	☐	☐

	Not At All Sure	Slightly Sure	Moderately Sure	Very Sure	Completely Sure
Changes in physical environment					
**How sure are you that you will…**					
25. … be able to get rid of all cigarettes from your environment this week?	☐	☐	☐	☐	☐
26. … not buy any cigarettes this week?	☐	☐	☐	☐	☐
27. … be able to substitute smoking time with something else this week?	☐	☐	☐	☐	☐
Emotional transformation					
**How sure are you that you can…**					
28. … direct your emotions/feelings to the goal of being smoke free every week?	☐	☐	☐	☐	☐
29. … motivate yourself to be smoke free every week?	☐	☐	☐	☐	☐
30. … overcome self-doubt in accomplishing the goal of being smoke free every week?	☐	☐	☐	☐	☐

	Not At All Sure	Slightly Sure	Moderately Sure	Very Sure	Completely Sure
Practice for change					
**How sure are you that you can…**					
31. … keep a self-diary to monitor your smoking urge every week?	☐	☐	☐	☐	☐
32. … be smoke free every week if you encounter barriers?	☐	☐	☐	☐	☐
33. … change your plan for being smoke free every week if you face difficulties?	☐	☐	☐	☐	☐

	Not At All Sure	Slightly Sure	Moderately Sure	Very Sure	Completely Sure
Changes in social environment					
**How sure are you that you can get the help of a…**					
34. …family member to be smoke free every week?	☐	☐	☐	☐	☐
35. …friend to be smoke free every week?	☐	☐	☐	☐	☐
36. …health professional to be smoke free every week?	☐	☐	☐	☐	☐

	Not At All Likely	Somewhat Likely	Moderately Likely	Very Likely	Completely Likely
Behavior change: Initiation					
**How likely is it that you will…**					
37. …quit smoking in the upcoming weeks.	☐	☐	☐	☐	☐
Behavior change: Sustenance					
**How likely is it that you will…**					
38. … smoke free every week from now on.	☐	☐	☐	☐	☐

Thank you for your time!

SCORING

Construct of advantages: Scale: Never (0), Hardly ever (1), Sometimes (2), Almost always (3), Always (4). Summative score of Items 10-14. Possible range: 0- 20. High score associated with likelihood of initiation of behavior change.

Construct of disadvantages: Scale: Never (0), Hardly ever (1), Sometimes (2), Almost always (3), Always (4). Summative score of Items 15-19. Possible range: 0- 20. Low score associated with likelihood of initiation of behavior change.

Subtract the score of disadvantages from advantages to come with a score for **participatory dialogue.**

Construct of behavioral confidence: Scale: Not at all sure (0), slightly sure (1), moderately sure (2), very sure (3), completely sure (4). Summative score of Items 20-24. Possible range 0-20. High score associated with likelihood of initiation of behavior change.

Construct of changes in physical environment: Scale: Not at all sure (0), slightly sure (1), moderately sure (2), very sure (3), completely sure (4). Summative score of Items 25-27. Possible range 0-12. High score associated with likelihood of initiation of behavior change.

Construct of emotional transformation: Scale: Not at all sure (0), slightly sure (1), moderately sure (2), very sure (3), completely sure (4). Summative score of Items 28-30. Possible range 0-12. High score associated with likelihood of sustenance of behavior change.

Construct of practice for change: Scale: Not at all sure (0), slightly sure (1), moderately sure (2), very sure (3), completely sure (4). Summative score of Items 31-33. Possible range 0-12. High score associated with likelihood of sustenance of behavior change.

Construct of changes in social environment: Scale: Not at all sure (0), slightly sure (1), moderately sure (2), very sure (3), completely sure (4). Summative score of Items 34-36. Possible range 0-12. High score associated with likelihood of sustenance of behavior change.

For **modeling** initiation dependent variable can be Item 37: not at all likely (0), somewhat likely (1), moderately likely (2), very likely (3), and completely likely (4) and multiple regression can be used. For modeling sustenance dependent variable can be Item 38: not at all likely (0), somewhat likely (1), moderately likely (2), very likely (3), and completely likely (4) and multiple regression can be used.
